# Ultrafast Spin Dynamics beyond *s*‑Wave Magnets: A Universal Polarization Dependence

**DOI:** 10.1021/acs.nanolett.5c04475

**Published:** 2025-10-23

**Authors:** Zhaobo Zhou, Junjie He

**Affiliations:** Faculty of Science, 37740 Charles University, Prague 12843, Czech Republic

**Keywords:** ultrafast spin dynamics, *s*-wave magnets, OISTR, altermagnets, real-time TDDFT

## Abstract

Symmetry and hybridization yield anisotropic but nodal-less Fermi surfaces in *s*-wave ferromagnets (FMs) and antiferromagnets (AFMs), while they produce distinct momentum-space nodes in altermagnets (AMs). Both drive anisotropic femtosecond magnetization dynamics, but this link remains little explored. Here, we investigate laser-driven ultrafast spin dynamics in FMs, AFMs, and AMs with varying polarization angles using time-dependent density functional theory. We demonstrated, in FMs and AFMs, that laser polarization controls the amplitude of anisotropic yet symmetric demagnetization. In contrast, AMs—featuring spin nodal structures—exhibit sublattice-asymmetric demagnetization that is highly sensitive to laser incidence. This behavior arises from the anisotropy of the Fermi surface and band dispersion, which governs optical-induced intersite spin transfer (OISTR). We proposed a unified framework using the band-path-resolved local density of states to understand anisotropic OISTR and its impact on spin dynamics. Our results establish a direct connection between polarization-dependent ultrafast spin responses and the anisotropic electronic structure of materials.

## Graphical abstract

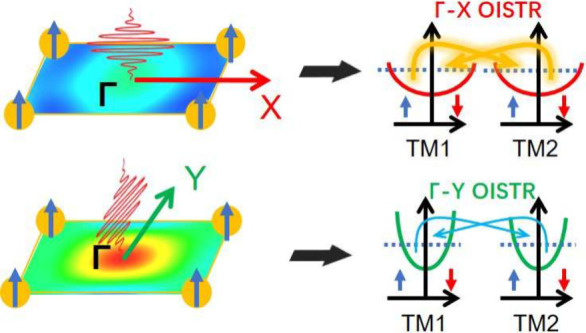

The discovery of ultrafast demagnetization in Ni in 1996 marked a milestone in femtomagnetism, revealing that the magnetic order of a solid can be quenched on subpicosecond time scales under intense femtosecond laser excitation.^1^ Since then, extensive experimental and theoretical efforts over the past three decades have greatly advanced our understanding of ultrafast spin dynamics in conventional ferromagnets (FMs) and antiferromagnets (AFMs).^2–8^ These studies have uncovered a variety of microscopic processes—such as spin–orbit-mediated spin-flip scattering,^8^ superdiffusive spin transport,^2–4^ and exchange-driven spin dynamics^7^—that contribute to the loss or redistribution of spin angular momentum at ultrashort time scales.

A recent theoretical breakthrough has identified optical-induced intersite spin transfer (OISTR) as a crucial mechanism governing laser-induced spin dynamics on femtosecond time scales.^9^ In OISTR, spin-polarized electrons are directly transferred between neighboring sublattice sites under the action of the laser field. This direct optical coupling between sites enables spin to be redistributed within a few femtoseconds, preceding any substantial influence of electron–phonon or electron–magnon interactions. The predictive power of this mechanism has been corroborated by numerous experimental studies employing techniques such as time- and spin-resolved photoemission,^10,11^ transient magneto-optical Kerr effect,^12–16^ and ultrafast X-ray magnetic circular dichroism.^17–19^ Together, these observations firmly establish OISTR as a fundamental building block of ultrafast spin dynamics in condensed matter systems.

While most studies of OISTR have assumed laser-induced, spin-selective charge transfer to occur in an essentially isotropic fashion under linearly polarized light in conventional magnets. However, the emergence of altermagnets (AMs)—magnetic systems hosting *d*/*g*/*i*-wave nodal electronic structures in momentum space—has revealed an entirely new playground for ultrafast spin physics.^20,21^ Their symmetry-protected nodal structures give rise to strongly momentum-dependent spin splittings and electronic dispersions.^22,23^ As a result, laser pulses with different polarization angles can selectively excite spin carriers along distinct crystallographic directions, leading to profoundly anisotropic optical responses and spin dynamics.^24–27^

By contrast, conventional FMs and AFMs are often classified as *s*-wave magnets with nodal-less electronic structures in momentum space.^28^ Nevertheless, in these systems, crystalline symmetry and hybridization with nonmagnetic elements generally distort the Fermi surface far from a simple sphere (Figure 1a,b). Such anisotropic Fermi surface geometries, together with path-dependent band dispersions, imply that anisotropic charge and spin transfer—and hence anisotropic OISTR—should, in principle, also manifest in *s*-wave magnets. Yet, to date, this possibility has remained largely unexplored, and a systematic framework for understanding the general mechanisms of OISTR in FMs, AFMs, and AMs is still lacking.

**Figure 1:**
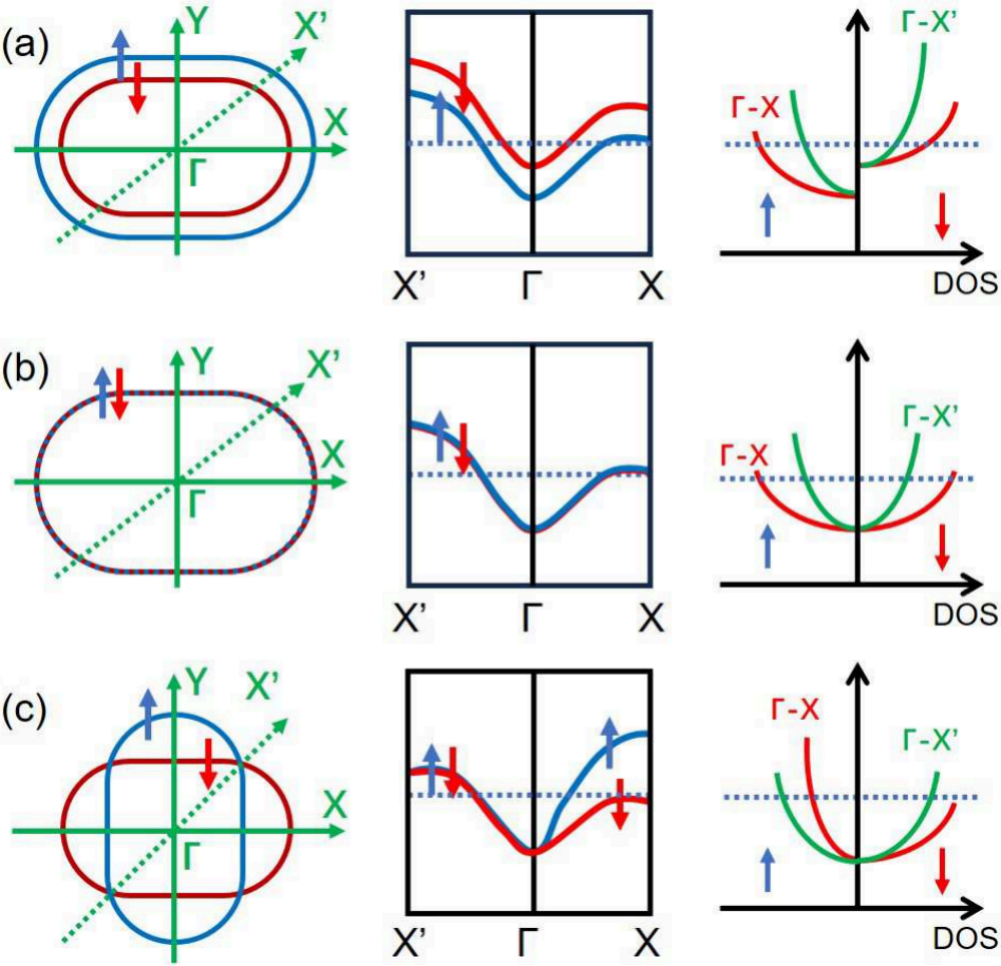
Constant-energy contour at Fermi surfaces, band dispersions, and path-resolved DOSs in a FM, a AFM, and a AM, respectively. Here, solid and dashed lines along the Γ–X and Γ–X′ paths represent the polarization direction of the laser.

Using time-dependent density functional theory (TDDFT), we systematically investigate ultrafast spin dynamics and the OISTR effect in FMs, AFMs, and AMs under laser pulses with varying polarization angles. We reveal that, for FMs and AFMs, the spin dynamics remain symmetrical due to their identical magnetic sublattices. However, their anisotropic electronic band structures lead to different electron excitation and magnitudes of demagnetization depending on the laser polarization. In contrast, AMs, due to the presence of nodal spin structures, exhibit asymmetrical spin dynamics together with a finite, polarization-dependent net magnetization. We further summarize the key similarities and differences in ultrafast spin dynamics and OISTR across these three classes of magnetic materials.

*General consideration for anisotropic OISTR*: Figure 1 compares the representative Fermi surfaces, band dispersions, and path-resolved densities of states (DOSs) for a FM, a AFM, and a AM along two distinct high-symmetry directions, Γ–X and Γ–X′. As shown in Figure 1a, the FM exhibits finite spin polarization throughout the Brillouin zone, reflected in a spin–split Fermi surface where the majority- and minority-spin sheets are clearly separated in momentum space. However, due to the underlying crystalline symmetry, the band dispersions along Γ–X and Γ–X′ are not identical, leading to noticeable differences in the DOS amplitude for the two paths, despite retaining a similar spin splitting in both cases.

In contrast, in a collinear AFM, the combination of time-reversal symmetry T and a half-lattice translation *T*_a_ forms an antiunitary symmetry $TT_{a}$ that leaves the Hamiltonian invariant, which enforces zero net magnetization. This symmetry enforces perfect spin degeneracy across the entire Brillouin zone (Figure 1b). This is directly visible in the Fermi surface as a complete overlap of the spin-up and spin-down sheets. Nevertheless, the dispersions along Γ–X and Γ–X′ still differ in shape because of the lattice symmetry, resulting in path-dependent DOS amplitudes. The DOS profiles for the two spin channels remain symmetric and mutually compensating, but with different overall magnitudes along the two directions.

AMs exhibit a qualitatively different scenario. Their momentum-dependent spin structure at the Fermi surface exhibits alternating spin splitting, which allows certain directions, such as the Γ–X path in Figure 1c, to display sizable spin polarization, whereas a symmetry-protected nodal plane enforces local spin degeneracy along the Γ–X′ path—resembling the AFM-like band structure. Strikingly, this character gives rise to a DOS that is spin-polarized along the Γ–X path but fully symmetric along the Γ–X′ path, a contrast absent in both conventional FMs and AFMs.

Having established the distinct band dispersions, Fermi-surface topologies, and path-resolved DOSs for FMs, AFMs, and AMs, we now turn to their implications for the OISTR effect by considering a laser pulse incident along the *z* axis (hereafter referred to as normal incidence). If the pulse is linearly polarized in the plane—specifically along either the Γ–X or Γ–X′ paths (Figure 2)—it can selectively excite electronic states residing predominantly along the corresponding high-symmetry paths. Due to the difference in the local density of states (LDOS) of the sublattice between the Γ–X and Γ–X′ paths, the resulting OISTR effect is, in principle, direction-dependent. In the following, we explicitly examine how such polarization-selective excitation manifests in FMs, AFMs, and AMs. We will analyze their ultrafast spin dynamics and microscopic mechanisms in these magnets.

**Figure 2:**
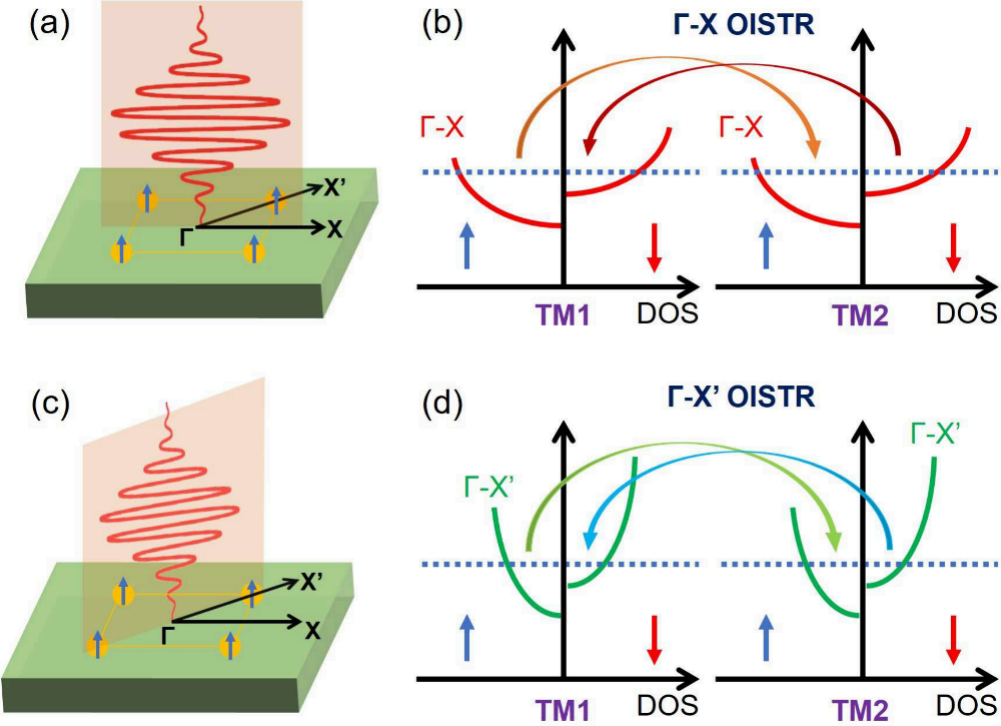
(a and c) Schematic of a linear-polarized laser incident along the *z* axis with in-plane polarization oriented along the Γ–X and Γ–X′ paths, respectively. (b and d) Corresponding schematic of the direction-dependent OISTR process. Two traditional metal sublattices are marked as TM1 and TM2, respectively. Red and blue arrows represent the spin-up and spin-down states, respectively.

*Ferromagnets*: We first employ the 2D FM monolayer CrSBr as a prototype platform to elucidate how distinct laser polarizations govern its OISTR and ultrafast demagnetization dynamics. Using TDDFT simulations, we examine two representative in-plane polarizations of laser pulses determined by intrinsic lattice symmetry. The laser pulses are characterized by a photon energy of 2.17 eV, a full width at half-maximum (fwhm) of 12 fs, and a fluence of 12.27 mJ/cm^2^.

Figure 3a presents the temporal evolution of the change in laser-induced magnetization, Δ*M*, for in-plane polarizations along the direction of Γ–X (magenta) and Γ–Y (olive) paths. During the first few femtoseconds, both polarizations produce nearly identical responses, reflecting early time electronic excitation that is largely insensitive to polarization. As the pulse evolves (≈10–20 fs), Δ*M* exhibits a rapid drop driven by OISTR, after which a clear polarization dependence emerges. For Γ–Y polarization, Δ*M* reaches nearly −0.4 μ_B_, indicating enhanced spin-transfer efficiency and coherent spin-charge dynamics. In contrast, Γ–X polarization results in a smaller reduction (≈−0.2 μ_B_).

**Figure 3:**
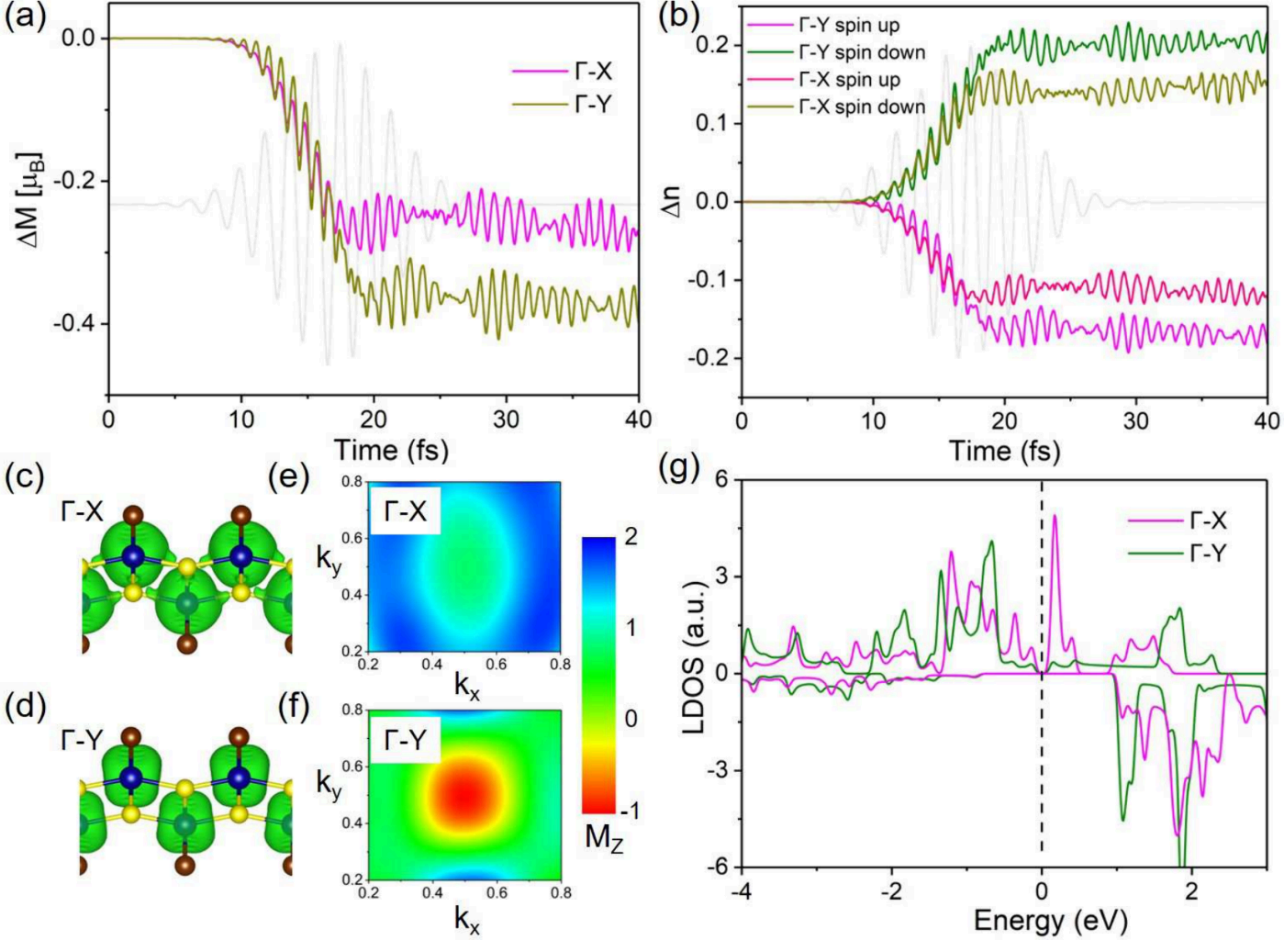
(a and b) Temporal evolution of the laser-induced change in the magnetization, Δ*M*, and the change in the spin-resolved charge, Δ*n*, of the Cr sublattice in FM CrSBr for in-plane polarizations along the Γ–X and Γ–Y paths. (c–f) Transient spin-resolved charge density and momentum-resolved magnetization maps at 35 fs under the polarized excitation along the Γ–X and Γ–Y paths, respectively. (g) Spin-resolved LDOS of a Cr sublattice along the Γ–X and Γ–Y paths.

Parts c and d of Figure 3 display the transient spin-resolved charge density at 35 fs under laser excitation along the Γ–X and Γ–Y paths, respectively. Γ–X polarization produces a more delocalized spin density in Cr sublattices, consistent with its weaker net spin transfer and reduced demagnetization. Conversely, Γ–Y polarization yields a more localized spin density on Cr atoms, in line with the stronger demagnetization observed in Figure 3a.

The momentum-resolved magnetization maps in Figure 3e,f further highlight this anisotropy. Γ–X polarization generates a pronounced out-of-plane magnetization hotspot centered at the Brillouin zone, whereas Γ–Y polarization produces an almost opposite-sign distribution with substantially weaker intensity. These results establish a direct link between laser polarization, momentum-space magnetization distribution, and anisotropic OISTR.

To uncover the microscopic origin of this anisotropy on demagnetization and OISTR, we examine the spin-resolved LDOS along the Γ–X and Γ–Y high-symmetry paths, as shown in Figure 3g. Near the Fermi level, the LDOS exhibits a pronounced asymmetric distribution between these two high-symmetry paths, arising from the distinct band dispersions of CrSBr along the Γ–X and Γ–Y paths. This dispersion anisotropy leads to a distorted Fermi surface, which, in turn, modulates the momentum-space excitation profiles (Figure 3e,f) and the efficiency of the OISTR process. Consequently, the demagnetization dynamics acquire a strong dependence on the laser polarization.

Additionally, Figure 3b displays the time-dependent variations in spin-resolved charge, Δ*n*, on the Cr sublattice in FM CrSBr, where Δ*n* ^*↑*^ and Δ*n* ^*↓*^ represent the spin-up and spin-down components, respectively. A clear polarization dependence emerges: different laser polarizations drive distinct spin-resolved charge transfer dynamics. In particular, under Γ–Y polarization, both Δ*n* ^*↑*^ and Δ*n* ^*↓*^ exhibit larger changes in amplitude compared to that with Γ–X polarization. Such direction-selective, spin-dependent charge flow generates an anisotropic spin current into the Cr sublattices, thereby accounting for the markedly different demagnetization efficiencies observed under the two excitation conditions.

*Antiferromagnets*: To uncover the polarization-dependent nature of ultrafast spin dynamics in AFMs, we focus on the prototypical AFM NiO. This material possesses well-defined crystalline symmetry and fully compensated spin structures. Figure 4a displays the time evolution of the local spin moment of a single Ni sublattice under two distinct laser polarization directions aligned with the Γ–M and Γ–X paths. These high-symmetry paths were chosen to reflect the intrinsic symmetry of NiO. In the early time regime (within the first 10–20 fs), both polarizations produce nearly identical demagnetization dynamics, indicating that the initial stage of electronic excitation is largely insensitive to the direction of the applied laser field—similar to what we observed for FMs. However, beyond ∼20 fs, the evolution diverges markedly: the Γ–X polarization drives a noticeably faster demagnetization and attains a larger overall magnitude than that of the Γ–M case. Quantitatively, the Γ–X case yields an additional reduction of approximately 0.2 μ_B_ per Ni sublattice, indicating a more efficient OISTR along this direction. This anisotropic response agrees with the transient spin-charge density variations shown in the inset of Figure 4a.

**Figure 4:**
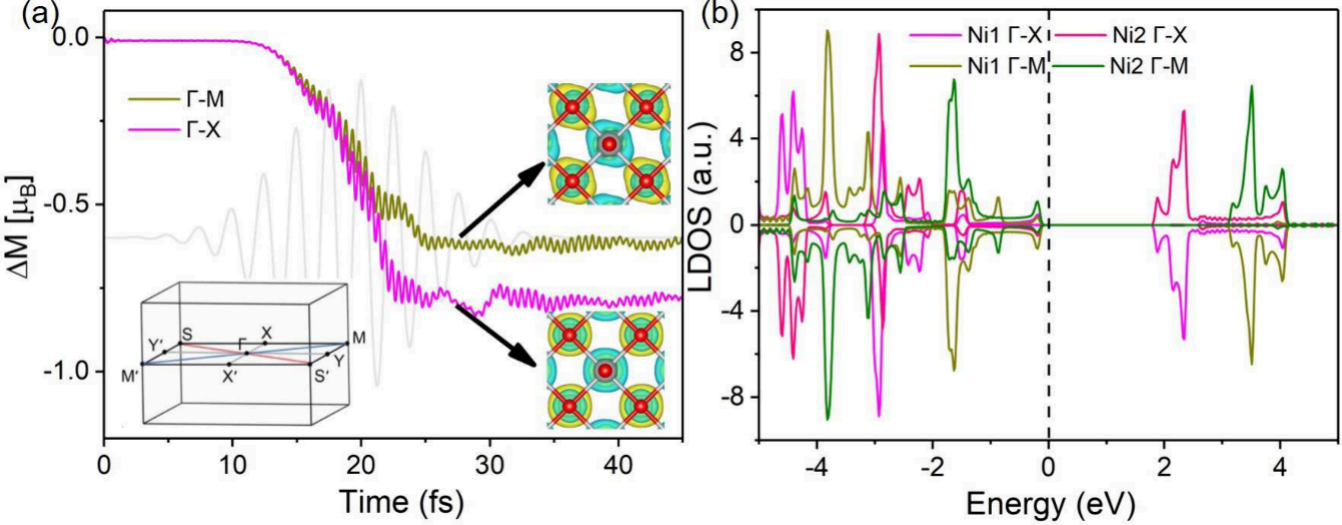
(a) Time evolution of the local spin moment of Ni sublattice in AFM NiO under two distinct laser polarization directions aligned with the Γ–M and Γ–X paths. Insets are the high-symmetry points in the Brillouin zone and the transient spin-resolved charge density at *t* = 26 fs. (b) Sspin-resolved LDOS of the Ni1 and Ni2 sublattices along the Γ–M and Γ–X paths.

Like in the FM case, the microscopic origin of this anisotropy can be traced to the spin-resolved LDOS of the Ni1 and Ni2 sublattices along the Γ–M and Γ–X paths, as shown in Figure 4b. For any given path, Ni1 and Ni2 consistently exhibit symmetric and perfectly compensated LDOS profiles, a direct manifestation of their antiparallel spin alignment. Remarkably, the LDOS characteristics differ significantly between the Γ–M and Γ–X paths. These variations directly influence the efficiency of OISTR: For the Γ–X case, the LDOS configuration promotes stronger intersite spin transfer, while for the Γ–M case, this process is comparatively hindered. This intrinsic electronic anisotropy naturally explains the observed difference in demagnetization between the two directions.

*Altermagnets*: To further investigate the polarization-dependent OISTR in AMs, we focus on two representative systems: KV_2_Se_2_O, a recently discovered planar *d*-wave AM,^29^ and CrSb, a typical bulk *g*-wave AM. We examine their laser-driven demagnetization dynamics under different light polarizations. For the *d*-wave AM KV_2_Se_2_O, the time evolution of the local spin moment of two V sites shows distinct dynamics behavior under laser incidence with two different polarization directions (Figure 5a): Two V sublattices undergo symmetric demagnetization along the direction aligned with the M−Γ–M′ path in the spin-degenerate nodal plane, preserving the zero net magnetization—similar to the local spin moment loss observed in AFMs. In contrast, when the laser is incident along the direction aligned with the Y−Γ–Y′ path in the spin-polarized region, the slight asymmetric demagnetization occurs between two V sublattices in the early time regime (12–20 fs). Beyond 20 fs, the asymmetric demagnetization becomes more pronounced, driving the KV_2_Se_2_O into a transient ferrimagnetic state with a magnetic moment of ∼0.1 μ_B_ per V atom. In addition, we also analyze the three components (M_*x*_, M_*y*_, and M_*z*_) of the transient spin moment of V atoms with spin–orbit coupling (Figure S1). It can be seen that only out-of-plane component (M_*z*_) exhibits a significant loss, whereas the in-plane components (M_*x*_ and M_*y*_) remain largely unchanged, indicating that no significant spin reorientation occurs during photoexcitation. A similar magnetization dynamics behavior is also observed in our previous work.^26^

**Figure 5:**
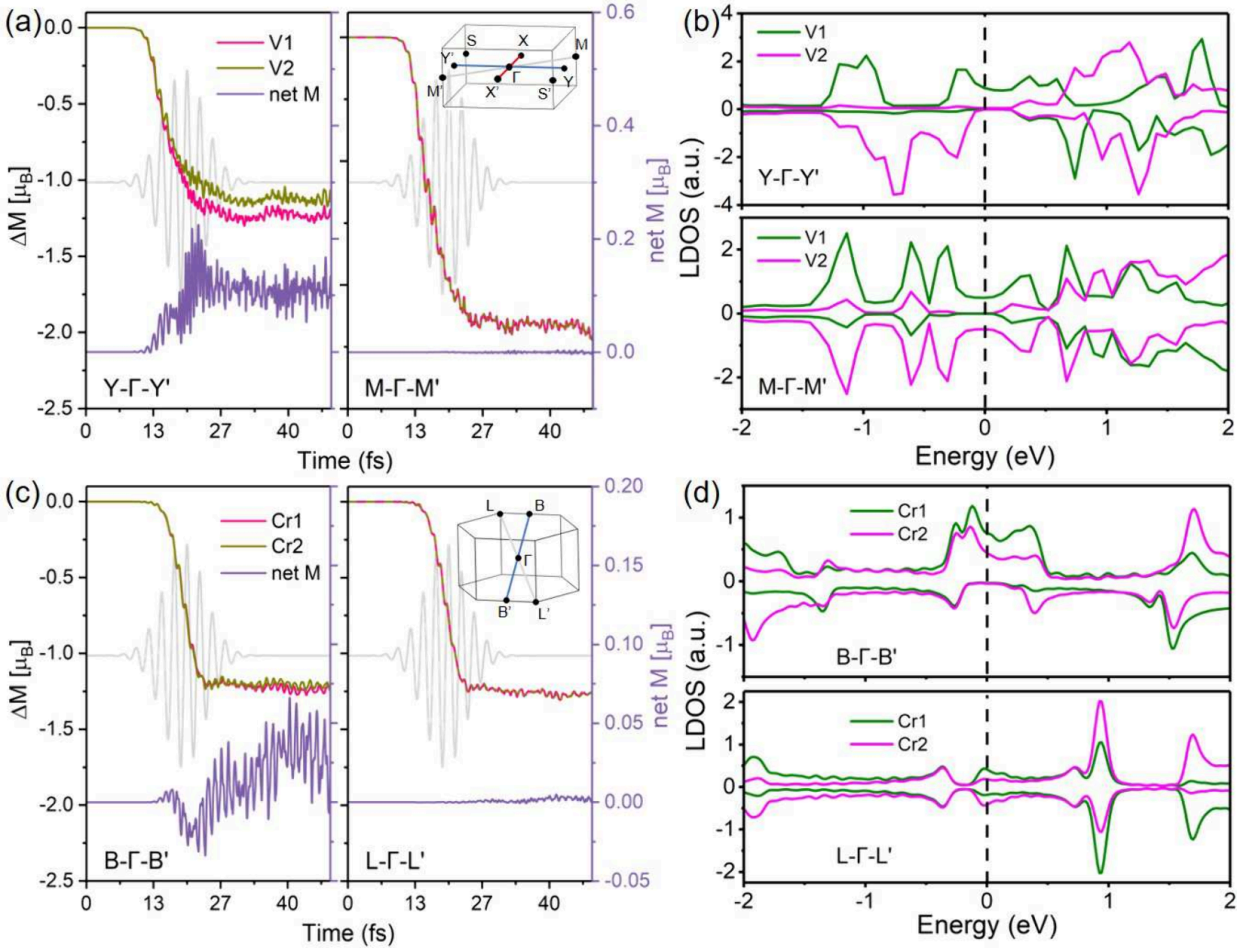
(a and c) Time evolution of the local moment of two V sublattices in KV_2_Se_2_O and two Cr sublattices in CrSb under two distinct laser incidence directions aligned with the Y−Γ–Y′ and M−Γ–M′ paths for the former and the B−Γ–B′ and L−Γ–L′ paths for the latter. (b and d) Spin-resolved LDOS of two V sublattices along the Y−Γ–Y′ and M−Γ–M′ paths in KV_2_Se_2_O and two Cr sublattices along the B−Γ–B′ and L−Γ–L′ paths in CrSb.

Notably, the asymmetric demagnetization observed in CrSb along the B−Γ–B′ path is off-plane-polarized, as the nodal structures of bulk *g*-wave AM are fully compensated for in the vertical and horizontal nodal planes of the Brillouin zone. In contrast, the spin structure of planar *d*-wave AM KV_2_Se_2_O is compensated for only in-plane, exhibiting an alternating pattern (Figure S2). These results verified the universality of polarization-dependent OISTR in AM.

Parts b and d of Figure 5 display the spin-resolved LDOS of the two magnetic sublattices—V1 and V2 in KV_2_Se_2_O and Cr1 and Cr2 in CrSb—along their respective momentum paths, providing microscopic insight into the OISTR process in AMs. The LDOS profiles fall into two distinct categories: asymmetric/uncompensated LDOS, found along the Y−Γ–Y′ path in KV_2_Se_2_O and the B−Γ–B′ path in CrSb, and symmetric/compensated LDOS, found along the M−Γ–M′ path in KV_2_Se_2_O and the L−Γ–L′ path in CrSb.

These results are consistent with our general principle for anisotropic OISTR: asymmetric (symmetric) LDOS gives rise to asymmetric (symmetric) demagnetization dynamics. Moreover, the larger LDOS weight on the V1 and Cr1 sublattices explains their stronger demagnetization amplitudes and the emergence of a transient net magnetization. We therefore conclude that in AMs, the spin dynamics and polarization-dependent OISTR response are critically governed by the band-path-resolved LDOS.

Our results establish a simple yet powerful framework to assess spin dynamics in magnetic materials directly from the band-path-resolved LDOS. If the LDOS of the magnetic sublattices remains symmetric along the laser incidence direction, the system exhibits symmetric spin dynamics and demagnetization of the sublattices. This is the case for conventional FMs and AFMs, where the sublattices are identical; in such systems, the demagnetization magnitude can be straightforwardly estimated from the LDOS weight. In contrast, an asymmetric LDOS distribution between sublattices gives rise to asymmetric demagnetization dynamics. This scenario naturally emerges in nodal magnetic systems, particularly in AMs, where nodal electronic structures may occur along both in-plane and out-of-plane crystallographic directions. Within this picture, one can rapidly predict whether a given laser incidence direction can induce significant spin transfer and demagnetization, and even anticipate the symmetry of the ensuing spin dynamics.

Experimentally, such effects could be probed by detecting the presence or absence of spin-polarized signals under varying laser polarizations. This concept finds an intriguing parallel in recent time-resolved magneto-optical Kerr effect (tr-MOKE) measurements on RuO_2_, where polarization-dependent signals were revealed in the *d*-wave AMs.^24^ Extending this methodology, one could envision similar polarization-resolved tr-MOKE or time-resolved X-ray magnetic circular dichroism (tr-XMCD) experiments to compare demagnetization amplitudes in FMs, AFMs, and *g*-wave AMs, thereby testing the predicted anisotropic OISTR effects.

It should be emphasized that our simulations focus exclusively on the early-time spin dynamics, prior to significant phonon involvement. At later time scales (*t* > 100 fs), phonon-mediated processes are expected to dominate demagnetization. An open and intriguing question is whether the anisotropic spin dynamics induced at early times by laser polarization can leave an imprint on the subsequent spin–phonon coupling pathways. If the initial electronic anisotropy modulates the efficiency of spin-to-phonon angular momentum transfer, it could manifest as a measurable polarization dependence in the amplitude or phase of coherent phonon oscillations. Such measurements, performed over a range of laser polarization angles, could provide a stringent test of the microscopic interplay between electronic anisotropy, ultrafast spin transfer, and lattice dynamics.

We have shown that the ultrafast spin dynamics of various magnetic materials under polarized laser excitation are governed by the interplay between laser polarization and the anisotropic electronic structure. For *s*-wave magnets (FMs and AFMs), identical sublattices lead to symmetric demagnetization, while the amplitude depends on the polarization direction. In contrast, nodal AMs exhibit intrinsic sublattice asymmetry in their demagnetization response. This behavior arises from the anisotropy of the Fermi surface and band dispersion, which governs the OISTR process. By introducing the band-path-resolved LDOS as a predictive tool, we establish a simple yet general framework for evaluating anisotropic OISTR effect and polarization-dependent spin dynamics in diverse magnetic systems. These insights pave the way for tailoring ultrafast spin control through the joint design of light polarization and material electronic structure.

Our study employs a fully noncollinear spin formulation of real-time TDDFT to investigate laser pulse-induced ultrafast spin dynamics in FMs, AFMs, and AMs. This approach incorporates electron interactions through the time-dependent Kohn–Sham (KS) equation, which describes noninteracting electrons evolving in an effective potential. The time-dependent KS equation is given by^30^

1 $$i\;\frac{\partial \psi_{j}\left(r,t\right)}{\partial t}=\{\frac{1}{2}{\left[-i\nabla -\frac{1}{c}[A\left(t\right)+A_{\mathrm{xc}}\left(t\right)]\right]}^{2}+v_{s}\left(r,t\right)\}\times \psi_{j}\left(r,t\right)$$

where ψ_*j*_ is a KS orbital and the effective KS potential *v*_s_(**r**,*t*) = *v*(**r**,*t*) + *v*_H_(**r**,*t*) + *v*_xc_(**r**,*t*) consists of the external potential *v*, the classical electrostatic Hartree potential *v*_H_, and the exchange-correlation (XC) potential *v*_xc_. The vector potential **A**(*t*) represents the applied laser field within the dipole approximation and **A**_xc_(*t*) the XC vector potential. We only propagate the electronic system while keeping the nuclei fixed.

All calculations were performed by real-time TDDFT as implemented through the full-potential augmented plane-wave ELK code.^31^ The real-time TDDFT simulations were conducted with a time step of Δ*t* = 2.4 as. All calculations adhered to the adiabatic local spin density approximation, consistent with methodologies established in our previous works.^32–35^

## Supplementary Material



## Notes

Three components (M_*x*_, M_*y*_, and M_*z*_) of the transient spin moment of the V atoms in KV_2_Se_2_O and spin structures of bulk *g*-wave AM and planar *d*-wave AM in the Brillouin zone (PDF)
