# Supplementary material for: Ultrafast Spin Dynamics beyond s‑Wave Magnets: A Universal Polarization Dependence
Source: Nano Lett. 2025 Oct 23;25(44):15978–84. doi: 10.1021/acs.nanolett.5c04475 (PMC12593343; doi:10.1021/acs.nanolett.5c04475)
Supplement: Supplementary file 1 [file nl5c04475_si_001.pdf]

Supporting Information for

Ultrafast Spin Dynamics Beyond *s*-Wave Magnets: a Universal Polarization Dependence

Zhaobo Zhou[1] and Junjie He[1,*]

[1]Faculty of Science, Charles University, Prague 12843, Czech Republic

Email: junjie.he@natur.cuni.cz

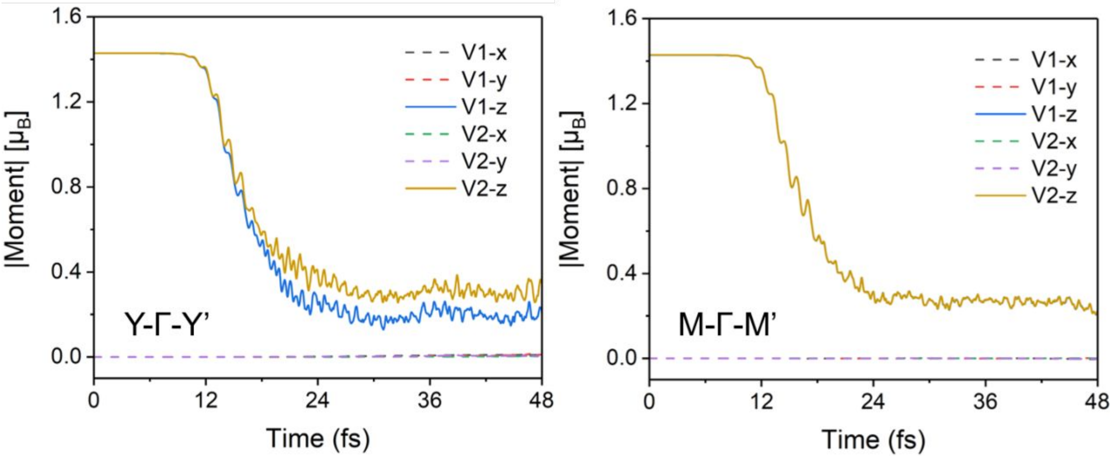

Figure S1. Evolution of the three components (Mx, My, and Mz) of the transient spin moment of V atoms with SOC during photoexcitation along Y-Γ-Y' and M-Γ-M' paths, respectively. The absolute values of the spin moments are taken to compare the moment change along three components.

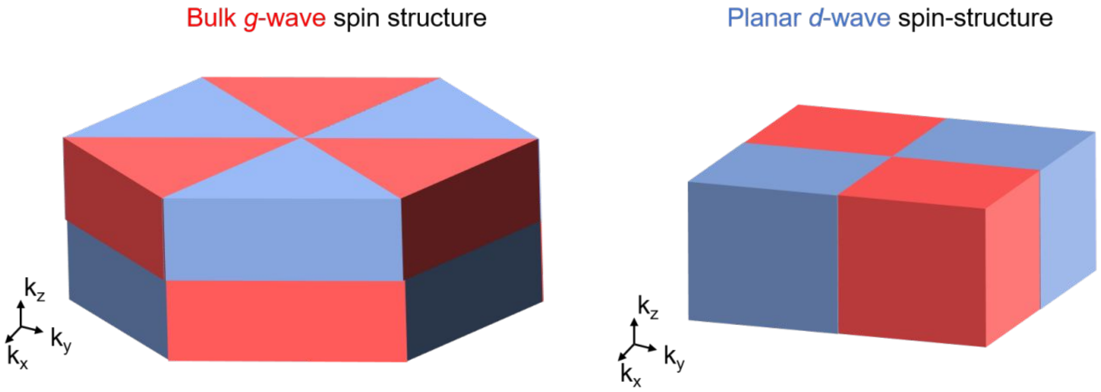

Figure S2. Spin structure of bulk *g*-wave AM and planar *d*-wave AM in the Brillouin zone.
